# Laparoscopic appendectomy and cholecystectomy in a 2-year-old with acute suppurative appendicitis and gallbladder torsion: a case report

**DOI:** 10.3389/fped.2025.1548318

**Published:** 2025-04-15

**Authors:** Jian Sun, You-Cheng Zhang, Chun-Hui Gu

**Affiliations:** Department of Pediatric Surgery, Huai'an Maternal and Child Health Hospital Affiliated to Yangzhou University, Huai'an City, Jiangsu Province, China

**Keywords:** appendectomy, appendicitis, cholecystectomy, gallbladder torsion, laparoscopy

## Abstract

**Background:**

Pediatric gallbladder torsion is a rare but potentially life-threatening cause of acute abdomen. The first case was reported in the 19th century, yet the precise pathogenesis remains unclear. Due to its nonspecific symptoms, gallbladder torsion in children is frequently misdiagnosed as more common conditions, such as acute appendicitis, cholecystitis, or gastrointestinal infections.

**Case presentation:**

A 2-year and 4-month-old boy was admitted to our emergency department with abdominal pain, fever, and vomiting. Initial blood tests revealed a white blood cell count of 13.13 × 10^9^/L, neutrophil percentage at 76.1%, and an absolute neutrophil count of 9.99 × 10^9^/L. Abdominal CT indicated thickened gallbladder walls, a partially blurred appendix margin, an intraluminal dense shadow, and multiple enlarged lymph nodes, along with pelvic effusion. A diagnosis of acute appendicitis was made. Emergency laparoscopy showed a suppurative appendix, leading to an appendectomy. Further inspection revealed a 360° counterclockwise torsion of the gallbladder neck with necrosis and black discoloration, without perforation. A laparoscopic cholecystectomy was performed. Nine days postoperatively, the child developed symptoms of an upper respiratory tract infection, necessitating transfer to the pediatric respiratory department, and was discharged on postoperative day 19 with no complications.

**Conclusion:**

Laparoscopic exploration for pediatric acute appendicitis should include the gallbladder as a standard investigation target.

## Introduction

Gallbladder torsion is a rare cause of acute abdomen in children, typically occurring in those with anatomical abnormalities of the gallbladder (1). The condition is primarily characterized by acute right upper quadrant pain, nausea, vomiting, and fever, which can easily be mistaken for other acute abdominal conditions such as acute appendicitis (2). If left untreated, it may result in gallbladder ischemia, necrosis, or even biliary peritonitis (1). Therefore, early recognition and prompt surgical intervention are critical for favorable outcomes. Here, we present a successful case of laparoscopic appendectomy and cholecystectomy in a child with acute suppurative appendicitis complicated by gallbladder torsion.

## Case report

**Figure 1:**
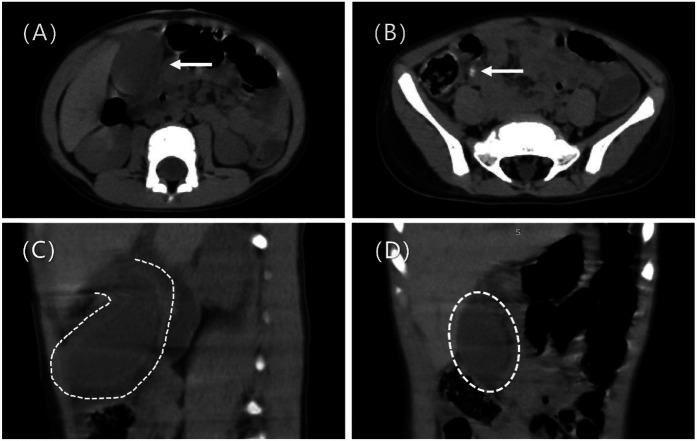
Shows preoperative imaging findings. **(A)** Preoperative CT axial scan shows thickening of the gallbladder wall and accumulation of fluid in the gallbladder (arrow). **(B)** Preoperative CT axial scan shows the local margin of the appendix is blurred, and a linear dense shadow is seen within the appendix lumen, suggestive of appendicolith (arrow). **(C)** Preoperative CT sagittal scan shows a floating gallbladder, with only the gallbladder neck connected to the liver (dashed line). **(D)** Preoperative CT coronal scan shows a distended, floating gallbladder (dashed line).

A 2-year and 4-month-old boy presented to the emergency department with abdominal pain, fever, and vomiting persisting for one day. On physical examination, the abdomen was soft with generalized tenderness, pronounced pain at McBurney's point, rebound tenderness, and muscle guarding in the right lower quadrant, without palpable masses. Laboratory findings included elevated white blood cells at 13,130/µl, a neutrophil percentage of 76.1%, and an absolute neutrophil count of 9,990/µl, while C-reactive protein levels were within normal limits. An abdominal CT scan showed no significant intra- or extrahepatic bile duct dilation, but it indeed shows thickening of the gallbladder wall, gallbladder fluid accumulation (Figure 1A), and suspected gallbladder torsion (Figures 1C,D), a partially indistinct appendix margin, an intraluminal dense shadow (Figure 1B), multiple enlarged lymph nodes, and pelvic effusion. The patient was admitted to the pediatric surgery department for suspected acute appendicitis.

**Figure 2:**
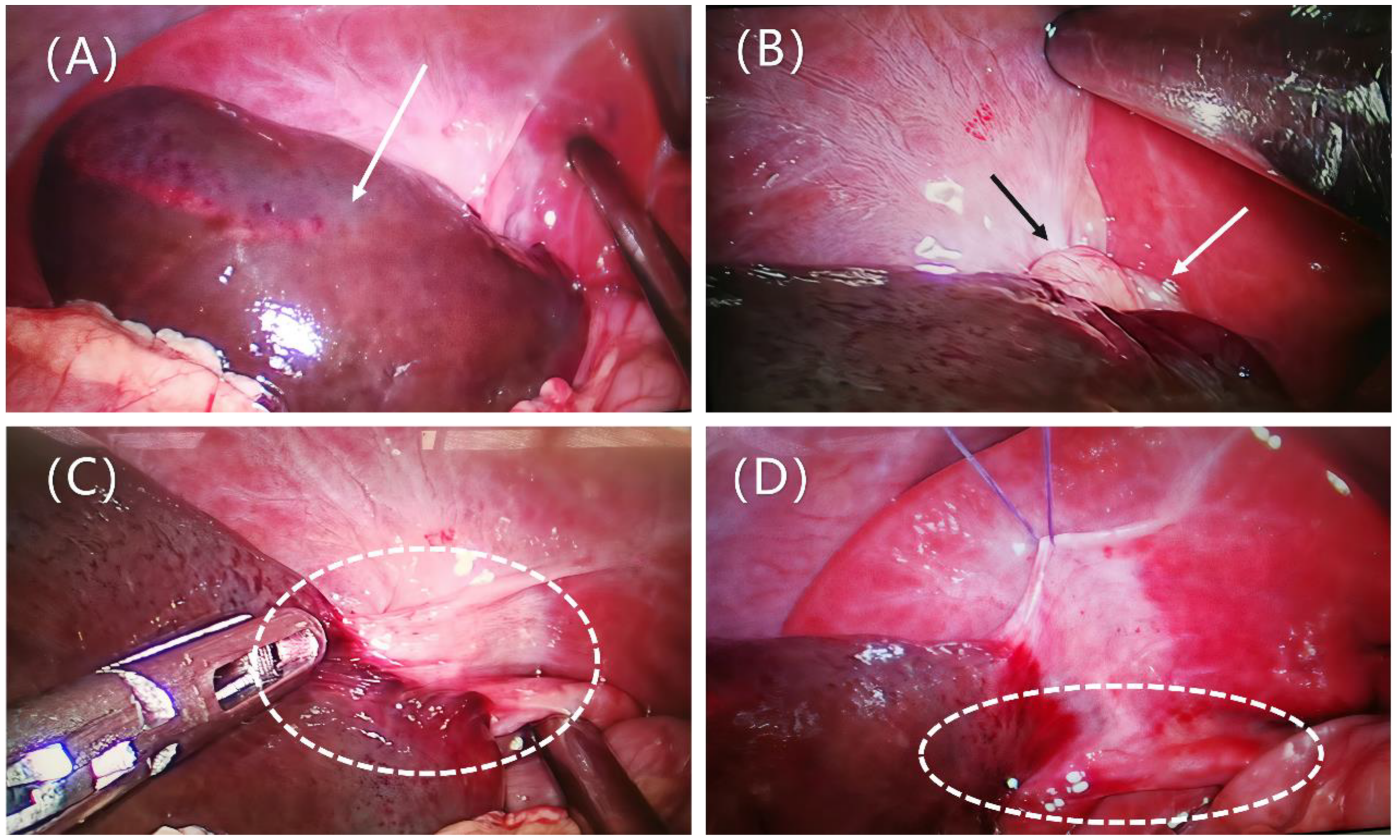
Presents intraoperative findings. **(A)** Distended gallbladder with blackened color (arrow). **(B)** Exposure of the gallbladder neck, showing the gallbladder twisted counterclockwise by 360° at the cystic duct and mesenteric level, with the mesentery (black arrow) and cystic duct (white arrow) twisted together. **(C)** The gallbladder is restored to its natural position by clockwise torsion, with a slight mesentery connection to the liver, classified as Type II according to Gross. The cystic duct is visible (dashed line). **(D)** The mesentery is suspended and unfolded; after 20 min, there is no color change in the gallbladder, and significant edema is observed in the cystic duct (dashed line).

**Figure 3:**
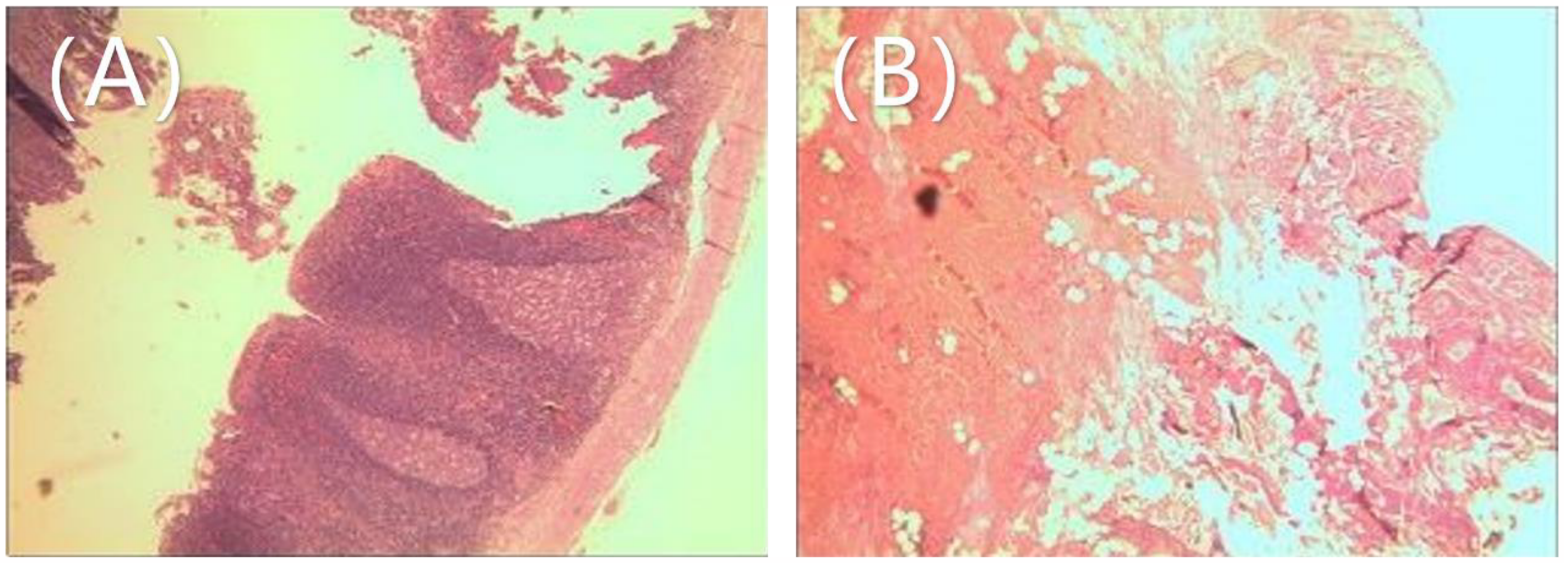
Postoperative pathological findings: **(A)** appendix: congestion and fibrinous purulent exudate on the serosal surface. **(B)** Gallbladder: Congestion, edema, and vascular dilation of the gallbladder wall, with infiltration of inflammatory cells.

Emergency laparoscopic surgery was performed through a 5 mm umbilical incision with a 5 mm trocar and camera. Two additional 5 mm incisions were made along the left rectus abdominis muscle and the midpoint between the umbilicus and the pubic symphysis. Standard laparoscopic appendectomy procedures for pediatric acute appendicitis were followed. The appendix was found to be congested, swollen, and covered with purulent exudate. Following the appendectomy, the entire small intestine was examined, revealing no significant abnormalities. The gallbladder, located in the subhepatic region, appeared blackened with severe edema and was twisted 360° counterclockwise at the cystic duct and mesentery level (Figures 2A,B). The gallbladder was restored to its natural position through clockwise torsion, and the mesentery was suspended and unfolded. After 20 min, no color change was observed in the gallbladder, and significant edema was noted in the cystic duct, indicating possible gallbladder necrosis (Figures 2C,D). The free mesentery was isolated, and the cystic artery and cystic duct were doubly ligated using Hem-o-Locks, followed by cholecystectomy. The procedure lasted 120 min, with minimal intraoperative blood loss. Postoperative histopathology revealed gallbladder wall congestion, edema, and vascular dilatation (Figure 3).

On the 5th day post-surgery, the child developed fever and cough, prompting a consultation with the pediatric respiratory department. A chest x-ray revealed right-sided pneumonia. The child was treated with antibiotics, nebulization, and antiviral therapy as symptomatic treatment. After three consecutive negative nucleic acid tests for the novel coronavirus, the child was transferred to the pediatric respiratory department on the 9th postoperative day. The child was discharged on the 19th postoperative day without any complications. Follow-up visits were conducted at one and three months post-surgery. Physical examinations and laboratory tests (including complete blood count and biochemical tests) showed no abnormalities.

## Discussion

Gallbladder Torsion is more commonly seen in the elderly and adult females. The earliest reported case of adult gallbladder torsion dates back to 1898, reported by the surgeon Wendel AV (3).Gallbladder torsion is an infrequent diagnosis in pediatric patients, typically occurring in those aged 1–17 years (4, 5), with the youngest reported patient being 1 years old (2, 6–11). The male-to-female ratio is approximately 11:4. Levard et al. (1) documented that out of 11 pediatric patients diagnosed with gallbladder torsion, 2 succumbed to misdiagnoses. The rarity and non-specific clinical manifestations contribute to the challenges in diagnosis. Common factors associated with gallbladder torsion include: (1) Congenital anatomical abnormalities: A floating gallbladder refers to a condition where the gallbladder is completely within the peritoneum, suspended in a floating state. The only mesentery attachment is at the gallbladder neck and cystic duct. The gallbladder duct is suspended below the liver, in a floating position (12, 13). Due to the high mobility of the gallbladder, it is prone to torsion along the axis of the cystic duct and the cystic artery (14). This case is of this type. (2) Acquired anatomical abnormalities: The incidence of gallbladder torsion is higher in the elderly, as the tissue structures around the gallbladder and the supporting fatty tissue gradually shrink with age. Tissue degeneration and reduced elasticity cause the gallbladder to droop, lengthening the mesentery (15, 16). Additionally, the motility of the stomach, duodenum, and transverse colon, as well as external factors such as physical labor and sudden changes in posture, can also induce gallbladder torsion (17). The acute onset of right upper quadrant pain is a common presenting symptom, often accompanied by tenderness or muscle guarding. Nausea and vomiting are frequently observed, while low-grade fever is possible but high-grade fever is uncommon. Laboratory tests typically show non-specific inflammatory markers, such as elevated white blood cell counts.

Conventional imaging techniques like ultrasonography and CT scans can assist in preoperative diagnosis, but most cases are diagnosed intraoperatively. CT and ultrasonography may show signs such as cystic duct torsion, abnormal gallbladder positioning, gallbladder wall thickening, and acalculous distension (7). If a floating gallbladder is detected on contrast-enhanced CT, with reduced blood flow towards the gallbladder wall, or the presence of the beak or spiral sign at the gallbladder neck, gallbladder torsion should be considered. MRI is considered the most valuable tool for preoperative confirmation, as it can clearly depict the anatomical relationship between the gallbladder and bile ducts, as well as the torsion axis (7). In cases where imaging results are inconclusive, laparoscopy can both diagnose and treat gallbladder torsion. The optimal timing for surgical intervention in gallbladder torsion is within two days of symptom onset; beyond this period, the mortality rate increases (18). Surgery is the definitive treatment (2, 10, 11), involving the detorsion of the gallbladder; if necrosis is present, cholecystectomy is required. For cases without evident necrosis, long-term follow-up data are needed to determine whether prophylactic cholecystectomy is warranted to prevent recurrence and complications.

Laparoscopic cholecystectomy has become the standard treatment in recent years due to its minimally invasive nature and rapid recovery. Reports indicate that laparoscopic procedures are both safe and effective in pediatric patients. Considering the risks of recurrence and mechanical stress injury, we do not advocate for the preservation of the gallbladder. In cases diagnosed with gallbladder torsion, we recommend cholecystectomy to prevent recurrence and to avert severe complications resulting from exacerbated cholangitis. We conducted a systematic search of English-language databases, including PubMed and Web of Science, to summarize pediatric gallbladder torsion cases, including our own, totaling 30 cases. Among these, 16 cases (including ours) were treated laparoscopically. Clinical data from these cases were also summarized, with detailed information provided in Tables 1, 2.

**Table 1:** Clinical data of 30 children with GT (1, 2, 4–7, 9, 11, 19–31).

Clinical data	Total	
Gender		
Male	22	73.33%
Female	8	26.67%
Mean age, years (range)		
Male	8.77	(2, 16)
Female	6.88	(1, 17)
Overall	8.2	(1, 17)
Clinical manifestations		
Vomiting	22	73.33%
Abdominal pain	30	100.00%
Fever	17	56.67%
Physical examination		
Right upper abdominal tenderness	28	93.33%
Muscle rigidity	15	50.00%
Poor mental status	7	23.33%
Abdominal distension	4	13.33%
Diminished bowel sounds	3	10.00%
Periumbilical tenderness	3	10.00%
Laboratory tests		
Elevated white blood cells	23	76.67%
Elevated CRP	9	30.00%
Elevated liver enzymes	6	20.00%
Imaging features		
Nonspecific		
Asymmetric gallbladder wall thickening	23	76.67%
Gallbladder enlargement	20	66.67%
Ascites	14	46.67%
Gallbladder hydrops	4	13.33%
Biliary dilation	2	6.67%
Gallstones	0	0.00%
Specific		
Horizontal long axis of gallbladder	6	20.00%
No blood flow signal in gallbladder wall	7	23.33%
Figure of eight sign	2	6.67%
Whirl sign	1	3.33%
Beak sign at gallbladder neck	5	16.67%
Site of torsion		
Duct	16	53.33%
Neck	8	26.67%
Body	2	6.67%
Not mentioned	4	13.33%
Angle of torsion		
Less than 180°	0	0%
180°	6	20%
More than 180°	19	63%
Not mentioned	5	17%
Surgical methods		
Open cholecystectomy	15	50.00%
Laparoscopic cholecystectomy	15	50.00%
Course prolongation	2	6.67%
GT caused by abdominal trauma	2	6.67%

Data are presented as *n* (%), unless stated otherwise. Prolonged course is defined as the time from onset to surgery exceeding one week.

**Table 2:** Cases of pediatric GT managed by laparoscopic cholecystectomy and with our patient added.

Author, year	Age (years)	Gender	Preoperative diagnosis	Time from onset to surgery (days)	Operation time (min)	Treatment for cystic duct	Complications	Postoperative hospital stay (days)
Kimura et al. (2)	11	M	GT	1	65	NA	None	4
Matsuda et al.	7	F	GT	2	NA	NA	None	3
Inoue et al. (8)	9	M	GT	4	NA	NA	None	4
Farnsworth et al. (26)	6	M	Appendicitis	1	NA	NA	None	NA
Musthafa et al. (25)	17	F	biliary colic	1	NA	Endoloop	None	2
Uemura et al. (11)	3	M	GT	3	104	Endoloop	None	6
Hoshi et al. (7)	5	M	GT	NA	NA	NA	None	NA
	13	M	GT	NA	NA	NA	None	NA
Kruger et al. (24)	16	M	Appendicitis	2	NA	NA	None	2
Lemons et al. (23)	12	M	Cholecystitis	3	NA	Hem-o-lok® clips	None	1
Nuyts et al. (20)	1	F	GT	2	65	Hem-o-lok® clips	None	3
Ren et al. (5)	6	F	GT	2	95	Non-Absorbable Suture (4–0)	None	6
Tadesse et al. (22)	7	M	GT	3	NA	NA	None	4
Tiep et al. (21)	5	F	GT	1 month	NA	NA	None	7
Yi Sun et al. (4)	13	M	Cholecystitis	11	99	Endoloop	None	4
Present case	2	M	GT,Appendicitis	0	120	Hem-o-lok® clips	None	19

F, female; M, male; NA, not available.

The three pediatric cases of gallbladder torsion complicated by acute appendicitis reported in this study share several notable clinical features. All patients presented with abdominal pain and vomiting, common symptoms of gastrointestinal distress. The first and third cases primarily involved right-sided abdominal pain, while the second case also featured nausea, vomiting, and worsened abdominal pain. Additionally, the second patient showed signs of lethargy and abdominal distension, likely due to progressing infection. None of the patients were initially diagnosed with gallbladder torsion, and the primary diagnosis in all cases was acute appendicitis. However, laparoscopic exploration revealed gallbladder torsion in each instance. The first patient did not undergo imaging prior to surgery, but intraoperative findings revealed a distended and twisted gallbladder, while the second case's preoperative CT scans suggested possible appendiceal perforation along with gallbladder distension. All patients underwent laparoscopic surgery, including appendectomy and cholecystectomy, with no significant postoperative complications, although one patient developed respiratory symptoms, which were successfully treated. These cases underscore the importance of considering gallbladder torsion in the differential diagnosis when managing pediatric patients with acute right-sided abdominal pain, particularly when conventional diagnoses like appendicitis do not fully explain the clinical presentation. Routine exploration of the gallbladder should be included in the surgical management of suspected appendicitis to avoid missed diagnoses and improve patient outcomes.

## Conclusion

This report describes a successful laparoscopic appendectomy and cholecystectomy in a 2-year-old boy with acute suppurative appendicitis and gallbladder torsion. Routine inspection of the gallbladder should be considered in cases of pediatric acute appendicitis. Although the clinical presentation of gallbladder torsion is non-specific, clinicians and radiologists should enhance their ability to recognize pediatric patients with right-sided abdominal pain to avoid misdiagnosis, especially when traditional diagnoses (such as appendicitis) do not fully explain the clinical manifestations.

## Data Availability

The raw data supporting the conclusions of this article will be made available by the authors, without undue reservation.

## References

[1] Levard G Weil D Barret D Barbier J. Torsion of the gallbladder in children. J Pediatr Surg. (1994) 29(4):569–70. 10.1016/0022-3468(94)90095-7 8014819

[2] Kimura T Yonekura T Yamauchi K Kosumi T Sasaki T Kamiyama M. Laparoscopic treatment of gallbladder volvulus: a pediatric case report and literature review. J Laparoendosc Adv Surg Tech A. (2008) 18(2):330–4. 10.1089/lap.2007.0057 18373471

[3] Mohamed Ali H Wahab ERA Damaj A Moussawi B Audi W Haidar M Intraoperative diagnosis of gallbladder volvulus. Case Rep Surg. (2023) 2023:1194077. 10.1155/2023/1194077 37941826 PMC10630001

[4] Sun Y Fang Z Cao X Zhang T Liu X Zhang J Pediatric gallbladder torsion managed by laparoscopic cholecystectomy: a case report and scoping review. Front Pediatr. (2024) 12:1506506. 10.3389/fped.2024.1506506 39877339 PMC11772262

[5] Ren H Liu H Liu X Wei H Tian P. Case report: rare floating gallbladder torsion in a child. Front Med (Lausanne). (2024) 11:1407716. 10.3389/fmed.2024.1407716 38873202 PMC11169684

[6] Hamada T Tajima Y Yamaguchi J Ueda T Izawa K Ohtani H Torsion of the gallbladder in a 3-year-old infant. J Hepatobiliary Pancreat Surg. (2009) 16(2):234–7. 10.1007/s00534-008-0027-9 19165413

[7] Hoshi R Uehara S Hosokawa T Kaneda H Koshinaga T. Gallbladder volvulus in two children: the importance of radiological features. Pediatr Int. (2022) 64(1):e15260. 10.1111/ped.15260 35938602

[8] Inoue S Odaka A Hashimoto D Tamura M Osada H: Gallbladder volvulus in a child with mild clinical presentation. Pediatr Radiol. (2011) 41(1):113–6. 10.1007/s00247-010-1753-0 20593170

[9] Kitagawa H Nakada K Enami T Yamaguchi T Kawaguchi F Nakada M Two cases of torsion of the gallbladder diagnosed preoperatively. J Pediatr Surg. (1997) 32(11):1567–9. 10.1016/S0022-3468(97)90454-1 9396527

[10] Nakao A Matsuda T Funabiki S Mori T Koguchi K Iwado T Gallbladder torsion: case report and review of 245 cases reported in the Japanese literature. J Hepatobiliary Pancreat Surg. (1999) 6(4):418–21. 10.1007/s005340050143 10664294

[11] Uemura S Maeda H Obatake M Namikawa T Kitagawa H Fujieda Y Laparoscopic cholecystectomy for gallbladder torsion in a 3-year-old child. Acute Med Surg. (2021) 8(1):e722. 10.1002/ams2.722 34987833 PMC8693826

[12] Copely K Dawkins A. The floating gallbladder. Abdom Radiol (NY). (2020) 45(10):3369–70. 10.1007/s00261-020-02420-x 31993698

[13] David M Mattia B Seda G Benjamin N Jerome H. Gallbladder torsion: a cholecystectomy that cannot be delayed. Acta Chir Belg. (2020) 120(4):279–81. 10.1080/00015458.2018.1553819 30633646

[14] Kalyanasundaram S Fernando S. Atypical presentation of cholecystitis with torsion of the gallbladder diagnosed preoperatively in an unusual location. BJR Case Rep. (2022) 8(1):20210141. 10.1259/bjrcr.20210141 35136647 PMC8803225

[15] Caliskan K Parlakgumus A Koc Z Nursal TZ. Acute torsion of the gallbladder: a case report. Cases J. (2009) 2:6641. 10.1186/1757-1626-2-6641 20181172 PMC2827113

[16] Lim LX Mahin HH Burnett D. Gallbladder volvulus: an unexpected "twist". Radiol Case Rep. (2022) 17(5):1755–9. 10.1016/j.radcr.2022.03.026 35355530 PMC8958462

[17] Krishna PS Somasekar RDR Sankar AS. Unexpected intraoperative finding of gallbladder torsion. J Surg Case Rep. (2021) 2021(12):rjab478. 10.1093/jscr/rjab478 34909163 PMC8666153

[18] Moser L Joliat GR Tabrizian P Di Mare L Petermann D Halkic N Gallbladder volvulus. Hepatobiliary Surg Nutr. (2021) 10(2):249–53. 10.21037/hbsn-20-771 33898569 PMC8050573

[19] Joshi M Mahalakshmi VN. Spontaneous gall bladder torsion with gangrene in a child: a rare case. Afr J Paediatr Surg. (2011) 8(2):262–3. 10.4103/0189-6725.86084 22005385

[20] Nuyts J Vanhoenacker C Vellemans J Aertsen M Miserez M. Gallbladder torsion: a rare cause of acute abdomen in a 12-month old child. Acta Chir Belg. (2024) 124(1):62–5. 10.1080/00015458.2023.2168073 36632772

[21] Tiep CM Ninh TP Hung ND Nga NTT Toan NM Hung PN Gallbladder volvulus in a 5-years old vietnamese female: a case report. Clin Case Rep. (2024) 12(4):e8743. 10.1002/ccr3.8743 38590331 PMC10999549

[22] Tadesse MM Sefu MS. Gallbladder volvulus in pediatric age: case report. Int J Surg Case Rep. (2024) 119:109664. 10.1016/j.ijscr.2024.109664 38688150 PMC11067458

[23] Lemons WJ Desimone R Seifarth FG. Hybrid single-port cholecystectomy of a pediatric gallbladder volvulus. Cureus. (2022) 14(4):e23801. 10.7759/cureus.23801 35518536 PMC9066953

[24] Kruger E Georgiou E. Gall bladder torsion masquerading as appendicitis in a teenage boy. S Afr J Surg. (2022) 60(4):319–20. 10.17159/2078-5151/SAJS3846 36477068

[25] Musthafa S Aftab Z Ali SM Khanna M. Gallbladder volvulus with segmental right liver lobe hypoplasia/atrophy: a preoperative diagnostic dilemma. BMJ Case Rep. (2018) 2018:bcr2018224474. 10.1136/bcr-2018-224474 29884666 PMC6011498

[26] Farnsworth TC Weiss CA III. Diagnosis and treatment of gallbladder torsion in a 6 year old. JSLS. (2013) 17(2):327–9. 10.4293/108680813X13654754534396 23925030 PMC3771803

[27] Tanaka S Kubota D Oba K Lee SH Yamamoto T Uenishi T Gallbladder torsion-induced emphysematous cholecystitis in a 16-year-old boy. J Hepatobiliary Pancreat Surg. (2007) 14(6):608–10. 10.1007/s00534-007-1211-z 18040631

[28] Narchi H Thomas M. Acute abdominal pain in a 6-year-old child. Eur J Pediatr. (1999) 158(11):943–5. 10.1007/s004310051248 10541955

[29] Koplewitz BZ Manson DE Ein SH. Posttraumatic torsion of accessory lobe of the liver and the gallbladder. Pediatr Radiol. (1999) 29(11):799–802. 10.1007/s002470050698 10552054

[30] Salman AB Yildirgan MI Celebi F. Posttraumatic gallbladder torsion in a child. J Pediatr Surg. (1996) 31(11):1586. 10.1016/s0022-3468(96)90187-6 8943132

[31] Komura J Yano H Tanaka Y Tsuru T. Torsion of the gallbladder in a thirteen year old boy–case report. Kurume Med J. (1993) 40(1):13–6. 10.2739/kurumemedj.40.13 8355473

